# Hepatitis C virus seroprevalence in pregnant women delivering live-born infants in North Thames, England in 2012

**DOI:** 10.1017/S0950268815001557

**Published:** 2015-07-16

**Authors:** M. CORTINA-BORJA, D. WILLIAMS, C. S. PECKHAM, H. BAILEY, C. THORNE

**Affiliations:** Population, Policy and Practice Programme, UCL Institute of Child Health, London, UK

**Keywords:** Hepatitis C, migration, pregnancy, seroprevalence, unlinked anonymous survey

## Abstract

To estimate HCV seroprevalence in subpopulations of women delivering live-born infants in the North Thames region in England in 2012, an unlinked anonymous (UA) cross-sectional survey of neonatal dried blood spot samples was conducted. Data were available from 31467 samples from live-born infants received by the North Thames screening laboratory. Thirty neonatal samples had HCV antibodies, corresponding to a maternal seroprevalence of 0·095% (95% confidence interval 0·067–0·136). Estimated HCV seroprevalences in women born in Eastern Europe, Southern Asia and the UK were 0·366%, 0·162% and 0·019%, respectively. For women born in Eastern Europe seroprevalence was highest in those aged around 27 years, while in women born in the UK and Asia-Pacific region, seroprevalence increased significantly with age. HCV seroprevalence in UK-born women whose infant's father was also UK-born was 0·016%. One of the 30 HCV-seropositive women was HIV-1 seropositive. Estimated HCV seroprevalence for women delivering live-born infants in North Thames in 2012 (0·095%) was significantly lower than that reported in an earlier UA survey in 1997–1998 (0·191%). Data indicate that the cohort of UK-born HCV-seropositive women is ageing and that, in this area of England, most perinatally HCV-exposed infants were born to women themselves born in Southern Asia or Eastern Europe.

## INTRODUCTION

Around 185 million people are infected with hepatitis C virus (HCV) worldwide [1]. Injecting drug use (IDU) accounts for most new HCV infections in Europe, while globally, unsafe blood supply and injections continue to contribute to the transmission of HCV in regions with the highest prevalence including Central, East and Southern Asia and North Africa/the Middle East (prevalence of 3·4–3·8%) [1]. In the UK, estimates derived from evidence synthesis indicate that around 214 000 people are chronically infected with HCV [2], with many remaining undiagnosed (an estimated 40% in London and 48% in Scotland) [3]. HCV-related hospital admissions and deaths from end-stage liver disease or liver cancer tripled between 1996 and 2010 [3].

The development of direct-acting antivirals (DAAs) has led to marked advances in treatment for HCV. Regimens which are shorter, interferon-free and with high (>95%) pangenotypic efficacy – including for cirrhotic and HIV co-infected patients – have raised for the first time the possibility of global targets for HCV elimination [4]. However, treatment rates in the UK have historically been low, with only around 3% of chronically infected individuals treated per year [5].

There are currently no effective interventions to prevent mother-to-child transmission (MTCT) of HCV (although rates may be reduced in HIV co-infected women with appropriate management) [6, 7] and understanding of the natural history of vertically acquired HCV disease is incomplete, particularly in adulthood [8]. In 2011, the UK National Screening Committee upheld their decision not to recommend routine screening for HCV in pregnancy, citing the scarcity of population-based data on contemporary antenatal HCV prevalence and other key evidence gaps, as well as the lack of effective interventions to prevent MTCT (http://www.screening.nhs.uk/hepatitisc-pregnancy); it is currently recommended that HCV testing is offered to pregnant women at increased risk of infection only. This recommendation is due to be reviewed in 2015 in light of updated evidence.

The last unbiased, population-based analysis of antenatal HCV seroprevalence in the North Thames region was in women delivering live-born infants between April 1997 and July 1998 [9]. Substantial demographic changes have since taken place; the proportion of live births in England and Wales to non-UK-born women increased from 16% in 2000 to 26% in 2012 overall, with an increasing number of deliveries to women born in Poland, Pakistan and India [10], and a doubling in the proportion of deliveries to women aged >35 years in this period [11]. An improved understanding of HCV epidemiology in the contemporary antenatal population is needed to inform screening and management strategies which reflect changing HCV treatment paradigms. The aim of this study was to establish HCV seroprevalence in relation to key demographic characteristics in a population of women delivering in North Thames in 2012.

## METHODS

### Data collection

The specimens analysed were collected as part of the unlinked anonymous (UA) survey of HIV infection in pregnant women, which was carried out using residual neonatal dried blood spot (DBS) samples routinely collected for metabolic newborn screening, usually at around age 1 week. Tests for HCV antibodies were conducted following testing for HIV-1 antibodies in the second quarter of 2012 (1 April–30 June) in the North Thames neonatal screening laboratory. The North Thames region comprises North London plus Bedfordshire, Hertfordshire and Essex, and accounted for about 17% of live births in England and Wales in 2012 [11]. The presence of HCV antibodies in the newborn infant reflects maternal infection status due to passive transfer of maternal antibodies *in utero*.

### Linking demographic data with neonatal DBS samples

The methods used by the UA survey on HIV in pregnant women, including anonymization and record-matching algorithms and procedures protecting against deductive disclosure, have been described in detail elsewhere [12]. All neonatal laboratory records corresponding to incoming neonatal screening cards received by the North Thames screening laboratory were downloaded into an extract file containing a unique laboratory number, child's date of birth and address. If the mother refuses all routine screening tests and/or anonymous testing, a blank spot is punched from the screening card. Fewer than 0·01% of mothers declined to have these tests performed for their infants. The laboratory number of the sample, and the plate and well position are stored in a file. This file was sent to the Centre for Infections, Public Health England (PHE) for further processing. After deletion of records from multiple births and repeat samples required for metabolic testing this file was matched to birth registration records at the Office for National Statistics (ONS) which provided information including hospital of birth, maternal borough of residence, and parental countries of birth. All patient-identifying information apart from laboratory number was then irreversibly deleted by the ONS before being returned to PHE. This demographic file was merged with the laboratory file using the unique laboratory number, which was subsequently deleted from the merged file before the samples were tested for HIV-1 and HCV antibodies. Serology results were merged with the demographic data using plate identifiers and well positions. At the end of the data-processing cycle all original data files were irreversibly deleted.

### Laboratory methods

DBS were eluted in PBS Tween-80 overnight at 4 °C and tested using the HCV Serodia particle agglutination test (Fujirebio Inc., Japan), which employs gelatin particles coated with HCV antigens and can detect HCV genotypes 1–4. This class of assays has previously been validated for detection of HCV antibodies in DBS achieving >95% sensitivity and specificity [13]. In our own validation using 16 HCV-antibody-positive DBS samples from asymptomatic, untreated adults, and 106 HCV-negative DBS samples from UK blood donors, 15 of the 16 HCV-positive samples were reactive, and all HCV-negative samples were non-reactive, yielding 94·1% sensitivity and 100% specificity. All positive results were repeated and tested with unsensitized particles to rule out non-specific reactions.

### Explanatory variables

We examined the association between estimated HCV seroprevalence and the following demographic risk factors: parental world regions of birth, maternal borough of residence, and maternal age. The United Nations classification of countries was used to categorize parental country of birth according to world geographical regions [14]; however, it should be noted that the Baltic states (Estonia, Latvia, Lithuania) were included in Eastern (not Northern) Europe, because of the epidemiological similarities between these and other former Soviet Union countries, with regards IDU-related risk factors for transmission of blood-borne viruses [15].

### Statistical analysis

Data were managed, and analyses were conducted using the R environment for statistical computing version 3.0.1 (CRAN, Austria). Fisher's exact tests were used for 2 × 2 comparisons. A logistic regression model with 2 d.f. spline terms on age as a continuous variable interacting with maternal region of birth was fitted.

### Ethical approval

Research ethical approval was granted by the East Midlands Research Ethics Committee (reference 12/EM/0488).

## RESULTS

Results from 31 467 non-repeat DBS specimens were analysed. Data linkage between birth registration records from the neonatal screening laboratory for North Thames and the ONS birth registration records was achieved for 31 316 (99·5%) DBS samples; for the 151 unlinked records, parental countries of birth were categorized as missing. There were 2299 (7·3%) infants born in the North Thames area whose mothers’ borough of residence was outside the survey area, although it is likely they lived close to it.

Thirty samples were HCV antibody positive [0·095%, 95% confidence interval (CI) 0·067–0·136], with seroprevalence varying by maternal region of birth (Table 1). HCV seroprevalence was low in UK-born women (0·019%) and highest in women born in Eastern Europe (0·366%) and the Asia-Pacific region (0·171%). Of the 26 HCV-seropositive women whose country of birth was known, three were born in the UK; the 11 women originating from Eastern Europe were born in Ukraine, Russia, Poland, Lithuania, the Czech Republic and Hungary, with the one woman from Southern Europe born in Italy. Of the six women originating from Southern Asia, four were born in Pakistan and two in India. One woman, born in Eastern Europe, had HIV co-infection, giving a prevalence of HIV/HCV co-infection in the population overall of 0·0032% (95% CI 0·0002–0·018).

**Table 1.** Neonatal anti-HCV prevalence by maternal region of birth, North Thames, England, 2012

Maternal region of birth	HepC−	HepC+	% total samples	Prevalence (%)
Africa	3188	1	10·14	0·031
Northern Africa	304	0	0·97	0·000
Western Africa	1154	0	3·67	0·000
Central Africa	252	1	0·80	0·395
Eastern Africa	1258	0	4·00	0·000
Southern Africa	220	0	0·70	0·000
UK	15 563	3	49·51	0·019
Rest of Europe	4480	12	14·29	0·267
Northern Europe	332	0	1·06	0·000
Western Europe	526	0	1·67	0·000
Eastern Europe	2997	11	9·57	0·366
Southern Europe	625	1	1·99	0·160
Americas	835	0	2·66	0·000
North America	286	0	0·91	0·000
Central America & Caribbean	275	0	0·87	0·000
South America	274	0	0·87	0·000
Asia-Pacific	5843	10	18·62	0·171
Western Asia	747	0	2·38	0·000
Central Asia	457	1	1·46	0·218
Southern Asia	3691	6	11·76	0·162
South Eastern Asia	388	1	1·24	0·257
Eastern Asia	327	1	1·04	0·305
Oceania	233	1	0·74	0·427
Not known	1528	4	4·87	0·261
Total	31 437	30	100·00	0·095

Table 2 shows anti-HCV prevalence stratified by maternal age in neonates of women born in the UK, Eastern Europe, and the Asia-Pacific region.

**Table 2.** Neonatal anti-HCV prevalence by maternal age: mothers born in the UK, Eastern Europe and Asia-Pacific and delivering in North Thames, England, 2012

	Maternal region of birth								
Maternal age (years)	UK			Eastern Europe			Asia-Pacific		
	HCV−	HCV+	Prev. (%)	HCV−	HCV+	Prev. (%)	HCV−	HCV+	Prev. (%)
<21	827	0	0·000	96	0	0·000	75	0	0·000
21–25	2180	0	0·000	461	1	0·217	835	0	0·000
26–30	3560	0	0·000	939	8	0·852	1634	2	0·122
31–35	3226	1	0·031	609	1	0·164	1245	2	0·161
>35	2945	2	0·068	350	1	0·286	1013	5	0·494
Total*	12 738	3	0·024	2455	11	0·448	4802	9	0·187

* Only women with known date of birth are included in this table.

**Fig. 1.**
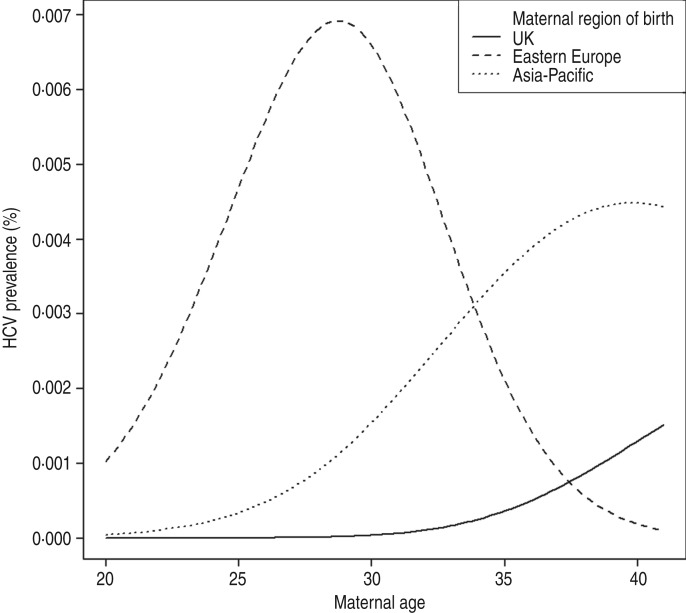
Estimated HCV seroprevalence by maternal age and region of birth, North Thames, England.

Seroprevalence was highest in Eastern European women aged 26–30 years (0·852%). There was a significant difference in age-adjusted seroprevalence between women born in the UK and in Eastern Europe (*P* < 0·001), and also between women born in the UK and the Asia-Pacific region (*P* = 0·044). A logistic regression model fitted with a 2 d.f. spline term in continuous maternal age showed a significant interaction (*P* = 0·033) between maternal age and region of birth, indicating a peak seroprevalence for women born in Eastern Europe and delivering in 2012 at around age 27 years, in contrast to a higher seroprevalence with increasing age in women born in the UK or the Asia-Pacific region (Fig. 1). Median maternal age in the HCV-seropositive women from Eastern Europe was 24 years (range 24–35), compared with 37 years in the UK/Southern Europe (range 33–41).

Seroprevalence was similar in outer (0·13%) and inner (0·10%) London (Table 3). Seroprevalence in metropolitan areas (inner and outer London) was almost twice that in non-metropolitan areas (0·116% and 0·053%, respectively), although this difference was not statistically significant (*P* = 0·15). Paternal region of birth was known for 22 of the 30 infants with seropositive samples and of these, nine (40·9%) fathers were born in the UK; however, for only two (9·1%) of the perinatally exposed infants were both parents UK-born. HCV seroprevalence in UK-born women whose infant's father was also UK-born was low, 0·016% (2/12 511).

**Table 3.** Neonatal anti-HCV prevalence by maternal borough of residence, North Thames, England, 2012

Borough	HepC−	HepC+	% total of samples	Prev. (%)
Inner London	8287	8	26·39	0·10
Outer London	11 486	15	36·58	0·13
Non-metropolitan	9367	5	29·81	0·05
Outside survey area*	2297	2	7·31	0·09
Total	31 437	30		0·10

* Considered with the North Thames population throughout our analyses.

## DISCUSSION

In this survey maternal HCV seroprevalence was 0·095% overall, with important differences between subpopulations. Our UA survey design of residual neonatal DBS is a robust method for reaching unbiased estimates of infection prevalence in pregnant women [9, 12], with a pan-genotypic assay which we validated for use with DBS. The very low proportion of mothers declining metabolic or further tests performed on their infants’ DBS ensures that refusal is not a source of bias. Prior to this study the last antenatal HCV seroprevalence data from England came from another UA survey carried out in 1997–1998 in the same region with similar methodology [9]. The overall seroprevalence obtained here is significantly lower (*P* < 0·001) than the 0·191% reported 15 years earlier (based on the seroprevalence estimates obtained with only the confirmed cases) [9]. HCV seroprevalence in UK-born women delivering in North Thames has declined significantly over the past 15 years, from 0·13% (95% CI 0·10–0·16) to 0·02% (95% CI 0·004–0·060). For UK-born women whose age was known, the age-adjusted seroprevalences between the two studies also differed significantly (*P* = 0·003), with these uniformly higher in women giving birth in 1997–1998. The antenatal HCV antibody prevalence in UK-born women in our study is just within the 95% credible interval (CrI) for women aged 15–59 years who have never injected drugs and are of white or other (non-South Asian) ethnicity living in England in a recent evidence synthesis analysis (seroprevalence 0·04%, 95% CrI 0·02–0·07) [3].

Seroprevalence in women born in the Asia-Pacific region showed a slight decline since the earlier study, from 0·22% to 0·17%, but this area of origin remained important with respect to higher HCV seroprevalence, consistent with the epidemiology of HCV in the UK [16, 17] and globally [1, 18]. Women born in Southern Europe had around a tenfold lower HCV seroprevalence compared to their counterparts in the late 1990s, from 1·58% to 0·16%, while seroprevalence in women born in Eastern Europe remained relatively stable (0·366% here *vs.* 0·40% in 1997–1998) [9]. By 2012, HCV seroprevalence in Eastern European-born women was 18 times higher than that in UK-born women. Consistent with national data, the overall proportion of births to UK-born women declined considerably between the 1997–1998 study (72%) and our study (50%) [9]; another noteworthy trend was the 19-fold increase in the proportion of deliveries to women born in Eastern Europe (from 0·5% to 19·2%). Trends in HCV seroprevalence in subpopulations and the shifting socio-demographic profile of pregnant women with HCV thus need consideration in the context of the changing patterns of births overall in the UK.

People who inject drugs (PWID) are important to consider when interpreting the epidemiology of HCV. An estimated 80–85% of individuals with chronic HCV infection in England are PWID [3, 19], with HCV prevalence of around 45% in current users, 30% in those with past use [3] and 18% in recent initiates [20]. The size of the population of PWID and ex-PWID is difficult to estimate reliably [21], particularly in pregnant women because of the perceived or real stigma associated with drug use [22]. Prevalence of current IDU was recently estimated as 0·65% in England and 0·79% in London, with around 3/1000 women estimated to be PWID [3]. A history of injecting drugs is more common in women originating from Central and Eastern Europe, with a survey of migrants living in London reporting that 2·5% of women were PWID [23], reflecting higher rates of IDU in Eastern *vs.* Western Europe [24]. HCV seropositivity rates in PWID are higher in Central and Eastern Europe than in the UK, with estimates of up to 85–94% in Lithuania, 66–83% in Romania, 61–73% in Ukraine and 49–96% in Russia [25, 26].

Data on HCV prevalence in contemporary pregnant women in Europe are scarce. In a large study in The Netherlands in 2003 in which around 4500 randomly selected samples from routine antenatal bloods were screened (half from women of non-Dutch origin), anti-HCV prevalence was 0·33% (95% CI 0·20–0·54), and lower in women of Western ethnicity (0·1%, 95% CI 0·04–0·34) than in those of non-Western ethnicity (0·6%, 95% CI 0·34–1·04) [27], as found here. In Ukraine, results from antenatal HCV screening in around 168 000 women in 2010 indicated a seroprevalence of 2·27% overall (Dr R. Malyuta, personal communication, June 2013), while a Russian study reported an antenatal HCV prevalence of 3% [28]. HCV seroprevalence was tenfold lower than this in women from Eastern Europe here, possibly reflecting the ‘healthy migrant effect’. In addition to a higher prevalence of IDU, risk factors for HCV acquisition in women born in Eastern Europe may include iatrogenic exposures and inadequately screened blood products.

Our finding of a low HIV co-infection rate concurs with the 1997–1998 study in which 2% of HCV-seropositive women had HIV co-infection [9]. Data from the UK indicate low rates of HCV co-infection in women living with HIV, with 1·9% of pregnant women in 2008–2010 and 4·6% of women receiving HIV care in 2000–2011 being anti-HCV positive [29] (S. Huntington, personal communication, March 2014). This reflects the very small proportion of pregnant women with HIV in the UK being PWID (e.g. 1·5% in 2007–2011) [30].

Population-based studies have demonstrated a birth cohort effect for HCV prevalence [1], e.g. with the ‘baby boomers’ born during 1945–1965 in the United States having a disproportionately high seroprevalence (3·25%), reflecting the peak in HCV incidence in the 1980s [31]. Here, there were no HCV infections in UK-born women aged <31 years, and those aged 31–35 years (born 1977–1981) had a seroprevalence of 0·031%, similar to the 0·02% seroprevalence in women aged <21 years in the 1997–1998 study born in the same period [9]. While seroprevalence increased with age in women born in the UK and Asia-Pacific, the peak seroprevalence in Eastern European women delivering in 2012 was at around 27 years.

In UK-born children, MTCT is the major mode of acquisition of HCV infection. Our findings suggest that there may be 120 infants born to HCV-positive mothers in North Thames annually. As the proportion of deliveries to women born outside of the UK is substantially higher in North Thames compared to England overall, further work is required to generalize these findings. To model the number of new vertical HCV infections occurring nationally, we therefore plan to estimate population-level antenatal HCV prevalence for England, based on the age- and region-of-origin-specific seroprevalence data obtained here. Our results may also inform estimates of HCV seroprevalence in women of child-bearing age in North Thames, which would need to account for different fertility rates in subpopulations at varying risk of HCV, e.g. PWID and migrants from countries with high HCV seroprevalence [32].

The HCV treatment pipeline is impressive and the development of short, all oral and highly efficacious regimens has changed the HCV treatment paradigm, although the pricing of currently licensed drugs is very high [33]. Although there is the potential to cure the majority of those treated, access to treatment is not universal and has become a topic of considerable global debate [34]. DAAs have yet to be licensed for paediatric treatment and their potential application in pregnancy (for maternal treatment and prevention of MTCT) has yet to be evaluated. The public health impact of preventing vertical transmissions through treatment of women with HCV prior to or potentially during pregnancy, curing HCV infection in early childhood, and of averting HCV-related sequelae in the women themselves is likely to be considerable.

Our study has some limitations. In around 0·05% of cases, linkage with birth registration data was not possible, because the baby was not registered or due to failure of the linkage process, resulting in some missing data. Although UA methodology reduced bias, the outcome was HCV seropositivity in the neonate (and thus in the mother), which does not equate to maternal chronic HCV infection given the potential for spontaneous viral clearance and HCV treatment. This approach was limited to women delivering live-born babies, and also precluded investigation of factors such as genotype, maternal disease status, infant outcomes and whether or not the women were aware of their HCV status. For 7% of the neonates sampled, the mother was resident outside of the North Thames area and although HCV seroprevalence was similar in this group to women living in North Thames, we were unable to characterize these women's borough of residence. The sensitivity and specificity to detect HCV seroprevalence from DBS samples found in our own validation and several other studies is high, and differences between our results and those of the 1997–1998 study on neonatal DBS in North Thames are commensurate with substantial demographic changes occurring in the intervening years.

An understanding of population-based anti-HCV prevalence is important for public health surveillance, determining screening policies and forecasting treatment need. This study provides contemporary antenatal HCV seroprevalence data and is the first population-wide study of HCV seroprevalence in women delivering live-born infants in England for over 15 years. Our results provide key data on antenatal HCV prevalence for consideration in the forthcoming re-assessment of the UK antenatal HCV screening policy, as well as providing updated estimates for inclusion in future evidence synthesis models for the UK as a whole.
